# Abortive Treatment of Bubo

**Published:** 1879-04

**Authors:** C. O. Weller

**Affiliations:** Columbus, Texas


### ABORTIVE TREATMENT OF BUBO.

By C. O. WELLER, M.D., Columbus, Texas.

At the meeting of the Texas State Medical Association at San Antonio, last April, Dr. Taylor, U. S. A., gave an account of the manner in which he treated buboes, when seen prior to the suppurative stage, which was so remarkable in its successful results as to make a decided impression on me.

His plan was this: to inject with a hypodermic syringe, into the inflamed gland, carbolic acid of the strength of from eight to twelve grains to the fluid-ounce of solvent. He had treated at that time, if I recollect correctly, some forty or more cases, some specific and others simple, with the uniform result of immediately stopping the inflammatory process, and establishing resolution. He claimed that after the operation, pain was soon relieved and never returned, and that he seldom had to make more than one injection. I determined to test this treatment the first opportunity that I should have. Soon a negro drayman who had a suppurating bubo in the left groin, that I had lanced sometime previously, came to me with a very large and painful swelling in the right groin.

I examined it carefully, and found that the suppurative stage had not begun, and injected with my hypodermic syringe, the point of which I carried some distance into the substance of the gland, a solution of carbolic acid, made by dissolving ten minims of the crystalized acid—previously rendered liquid by heat—in two drachms of glycerine, to which was added six drachms of water. The pain from the puncture was a little severe, but soon subsided, and in a day or two ceased altogether. Resolution was soon accomplished. A few weeks ago a white man came to me with a large bubo in the right groin. I injected about 25 *m* of the above solution of carbolic acid into it. This was about 7 o'clock P.M. The pain was considerable, and continued so the greater part of that night, but ceased the next day never to return. This patient made a speedy recovery. The result of treatment in my two cases, taken in connection with Dr. Taylor's forty cases, so convinces me of the value of carbolic acid in arresting the inflammatory process in the lymphatic glandular system, that I shall certainly continue to use it until something better is discovered.—*N. O. Med. and Surg. Journal.*

